# Influence of Specimen Width on Crack Propagation Process in Lightly Reinforced Concrete Beams

**DOI:** 10.3390/ma17225586

**Published:** 2024-11-15

**Authors:** Hongwei Wang, Hui Jin, Zhimin Wu, Baoping Zou, Wang Zhang

**Affiliations:** 1 School of Civil Engineering and Architecture, Zhejiang University of Science and Technology, Hangzhou 310023, China; wanghongwei@zust.edu.cn (H.W.); jinhui@zust.edu.cn (H.J.); wuzhimin@dlut.edu.cn (Z.W.); 2 State Key Laboratory of Hydroscience and Engineering, Tsinghua University, Beijing 100084, China; 3 State Key Laboratory of Coastal and Offshore Engineering, Dalian University of Technology, Dalian 116024, China; zwcivil@mail.dlut.edu.cn

**Keywords:** lightly reinforced concrete, crack propagation, three-dimensional, bond-slip

## Abstract

Models used to study the fracture process of concrete are often considered 2D, ignoring the influence of specimen width. However, during the fracture process in pre-cracked concrete beams, the crack length varies along the thickness direction, especially in reinforced concrete. To study the influence of specimen width on reinforced concrete fracture behavior, a 3D numerical method was used to simulate the crack propagation processes of lightly reinforced concrete beams based on Fracture Mechanics. Nonlinear spring elements with different stress-displacement constitutive laws were employed to characterize the softening behavior of concrete and the bond-slip behavior between the steel bars and concrete, respectively. It is assumed that the crack begins to propagate when the maximum stress intensity factor at the crack tip along the beam width reaches the initial fracture toughness of concrete. To verify the validity of the proposed method, the completed crack propagation processes of lightly reinforced concrete three-point bending notched beams were simulated, and the calculated load-crack mouth opening displacement curves showed a reasonable agreement with the experimental data. Moreover, the impact of the 2D reinforced concrete beam model on the crack propagation process was analyzed. The results indicate that at the initial loading stage, the external load P obtained from the 2D model is significantly larger than the result from the presented 3D model.

## 1. Introduction

The quasi-brittle materials such as concrete possess very little tensile strength, thus resulting in a relatively weak material under tension, and the concrete structures are usually reinforced at least in the area where tensile stresses are expected. Nevertheless, the concrete tensile strength is typically disregarded in both design and numerical analysis of reinforced concrete (RC) structures since its contribution to the ultimate capacity of the overall structure can be negligible [1]. Therefore, to obtain reliable analytical and numerical results for analyzing the damage processes, including cracking and instabilities phenomena [2,3,4], the cohesive behavior in the fracture process zone (FPZ) of concrete must be correctly simulated. For instance, in lightly reinforced concrete beams under three-point bending, the cracking moment may be larger than the ultimate capacity [5]. To comprehensively grasp the restraining impact of steel bars on concrete fracture behavior, analyzing the fracture process in lightly RC beams serves as a direct and effective approach.

The analytical methods have been widely explored to study the fracture process of lightly RC beams based on the assumption of plane deformed sections or the fracture theory. Gerstle et al. [6] and Ulfkjaer et al. [7] addressed the non-linearity of concrete by including strain-softening in a fictitious crack model, and the specimen response was determined analytically based on the assumption of plane deformed sections. Nevertheless, the strain-softening behaviors are not clearly related to the actual properties of the materials. Cadamuro et al. [8], Yang [9], and Yi [10] used more accurate approaches that take the softening constitutive relationship of concrete and the reinforcement stress-crack opening relationship obtained by means of preliminary studies carried out on the interaction between the reinforcing bar and the surrounding concrete into account. The calculated results were in good agreement with the experimental results. Fayyad and Lees [11] analyzed the crack propagation processes of lightly RC beams based on the plane-section assumption by considering the tensile softening behavior of concrete, bonding behavior between steel bars and concrete, and hardening behavior of steel bars. However, it has been shown that the plane-section assumption is no longer valid in the presence of bonding-slip behavior between steel bars and concrete [12]. Mi et al. [13] proposed an analytical method for the fracture processes of RC beams with a single crack based on $K_{\mathrm{IC}}^{\mathrm{ini}}$-stress intensity factor (SIF) crack propagation criterion, assuming complete bonding between steel bars and concrete. However, neglecting the bonding-slip behavior between steel bars and concrete cannot accurately reflect the actual influence of steel bars on the crack propagation process. Ruiz [14] and Ruiz et al. [15] proposed an effective slip length model to solve the propagation of a cohesive crack through a reinforcement layer, in which the reinforcement is represented by means of a free-slip bar bridging the cracked section, anchored at both sides of the crack at a certain distance, and the crack initiates when the maximum principal tensile stress reaches the tensile strength of concrete. Fan [16] simulated the fracture processes of lightly RC beams based on the nil-SIF crack propagation criterion, and an approximate relation between the stress of the steel bars and the concrete crack width at the steel bars [17] was adopted to simulate the bond slip between the steel bars and concrete. Wang et al. [12] improved Fan’s [16] simulation by considering the bonding-slip of the steel-concrete interface and the yielding behavior of steel bars. However, this method did not consider the closure effect on the crack opening displacement induced by the concentrated force of steel bars acting on the crack faces, and previous studies [18] have shown that the $K_{\mathrm{IC}}^{\mathrm{ini}}$-SIF criterion can more accurately describe the entire crack propagation process of concrete, especially for high-strength concrete. Most of these theoretical studies incorporate the reinforcement according to the principle of superposition by considering concrete fracture and adding the restraining effect of the reinforcement as a closing concentrated force into a two-dimensional (2D) fracture model [19]. Nevertheless, the actual RC beam is three-dimensional (3D). In addition, the SIFs at the crack tip are unevenly distributed along the specimen width, showing a trend of being large in the middle and small on both sides [20]. That is to say, the crack propagates from inside to outside along the specimen width. Therefore, the fracture process would be inconsistent with the actual situation if the 3D RC beam is simplified into a 2D model.

In most numerical methods, the fracture processes of lightly RC beams were simulated by adopting the stress-based crack propagation within a finite element framework. Yu et al. [21,22] combined a modified dynamic relaxation (DR) method with a perfect-plastic bond-slip law between steel bars and concrete to simulate the fracture process of RC beams subjected to static loading by using a 3D finite element model. This study found that smooth re-bars debond very quickly, even before the maximum load is attained, while the ribbed bars sew the main crack and force the surrounding material to protrude from the main crack surface. Lin et al. [23] and Wei et al. [24] analyzed the entire fracture process, including initiation, propagation, and failure for lightly RC beams with different reinforcement ratios, by employing the finite element method, assuming that the node breaks when the stress of the concrete element reaches the tensile strength of concrete. In this model, a virtual spring representing the action of cohesive force, which follows the linear softening relationship of concrete, was set up at each node in FPZ, and a pair of hypothetical concentrated forces representing the effect of steel bars on concrete were set up at the intersections of steel bars and crack faces. Ru et al. [25] studied the crack propagation process of 3D RC beams using the maximum circumferential stress criterion and the extended finite element method. This study used a linear law to represent the softening behavior of concrete and fixed bonding between steel bars and surrounding concrete. Ramos et al. [26] simulated the evolution of a crack in an RC beam using the virtual crack closure technique (VCCT) within a finite element framework. In this procedure, the debonding behavior of steel bars was not considered, and the crack starts to propagate when the stress in concrete exceeds the tensile strength of concrete. However, a very fine division on the element mesh is required to obtain the node stress with accuracy by adopting the stress-based crack propagation [27]. Therefore, it is necessary to develop a 3D finite element method to simulate the fracture process of RC based on the SIF-based crack propagation criterion.

In this paper, a 3D numerical method is proposed for simulating the crack propagation process in RC based on the nonlinear Fracture Mechanics Theory and the $K_{\mathrm{IC}}^{\mathrm{ini}}$-SIF crack propagation criterion. Different nonlinear spring elements are incorporated in the FPZ of concrete and the steel bar-concrete interface to characterize the softening behavior of concrete and the bond-slip behavior of the steel bar-concrete interface, respectively. The numerical method is then utilized to calculate the crack propagation processes in lightly RC beams subjected to three-point bending with initial cracks, and the computed load-crack mouth opening displacement (*P*-*CMOD*) curves are compared with experimental data to validate its effectiveness. Furthermore, the shear stress distribution along the steel bar-concrete interface during the cracking process is analyzed, and the influence of specimen width on the crack propagation process in lightly RC beams is discussed.

## 2. Numerical Program

**Figure 1:**
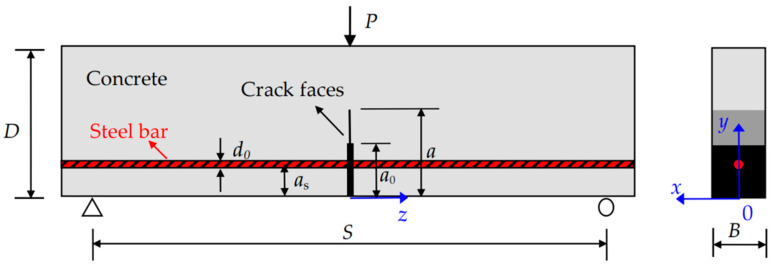
Lightly RC beam under three-point bending.

As illustrated in Figure 1, a notched lightly RC beam with a single layer of reinforcement subjected to static loading is considered herein, where *P* is the external load, *D*, *S*, and *B* are the height, span, and width of the beam, respectively, *a* is the crack length, *a*_0_ is the initial crack length, *a*_s_ is the thickness of the concrete cover, *d*_0_ is the steel bars diameter, and *x*, *y*, and *z* are the coordinates from the beam bottom center along the width, the height, and the span, respectively. The general finite element (FE) software ANSYS 15.0 is employed to numerically simulate the crack propagation process of the presented RC beam. The specific numerical procedure of the fracture process is described in the following sections: (1) material models and elements, (2) numerical modeling, and (3) numerical process.

### 2.1. Material Models and Elements

#### 2.1.1. Concrete

**Figure 2:**
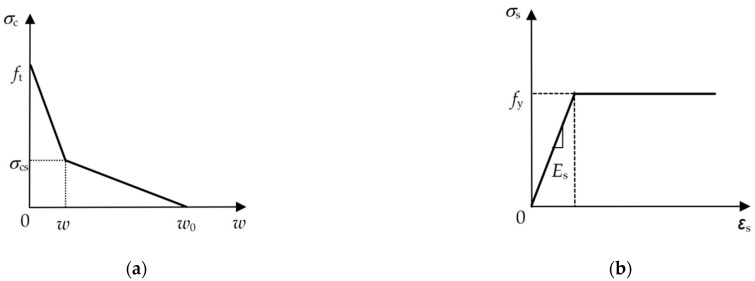
Constitutive relationships: (**a**) concrete and (**b**) steel bars.

The compressive strength of concrete is much larger than its tensile strength, and in the cracking process of a lightly RC beam, the damage to concrete only occurs by tensile failure. Therefore, based on the fictitious crack model, concrete is assumed to be an isotropic linear elastic material, and cohesive stresses acting on the fictitious crack faces are indicated to describe the softening behavior of concrete. As depicted in Figure 2a, a bilinear constitutive relationship, which has been well recognized as a simple and reasonable approximation of the softening curve of concrete [28,29,30,31], is utilized to calculate the cohesive stress *σ*_c_ acting on the surface of FPZ and can be written as

(1) $$\sigma_{c}=\left\{\begin{array}{cc} f_{t}-\left(f_{t}-\sigma_{\mathrm{cs}}\right)\left(w/w_{s}\right), & 0\leq w\leq w_{s}\\ \sigma_{\mathrm{cs}}\left(w_{0}-w\right)/\left(w_{0}-w_{s}\right), & w_{s}<w\leq w_{0}\\ 0, & w>w_{0} \end{array}\right.$$

where *f*_t_ is the tensile strength of concrete, parameters *w*_s_, *w*_0_, and *σ*_cs_ are in terms of the fracture energy of concrete *G*_F_ and *f*_t_, which can be expressed as [32].

(2) $$\begin{array}{ccc} w_{s}=0.8G_{F}/f_{t}, & w_{0}=3.6G_{F}/f_{t}, & \sigma_{\mathrm{cs}}=f_{t}/3 \end{array}.$$

The element SOLID 95 provided by ANSYS version 15.0 FE code, which is a 3D 20-node element with compatible displacement shape and is well suited to model curved boundaries, was employed to model the concrete behavior outside the FPZ in the numerical simulation. To reflect the nonlinear characteristics of concrete, a series of nonlinear spring elements were established at the nodes along the fictitious crack faces. The mentioned spring elements are virtual spring elements with no geometric dimensions or masses. Therefore, the unidirectional element COMBIN39, which has longitudinal capability in 3D application and nonlinear generalized force-deflection capability, can be utilized herein. The element COMBIN39 has large displacement capability for which there can be two or three degrees of freedom at each node, and it is defined by two preferably coincident node points and a generalized force-deflection curve considering the mechanical characteristics of the material. Once the crack propagates, the nodes with the same coordinates on the fictitious crack faces are connected using COMBIN39 elements, and the force-deflection curve can be obtained by the softening constitutive relationship as shown in Equations (1) and (2).

#### 2.1.2. Reinforcing Steel Bars

An elastic-perfectly plastic constitutive law, as shown in Figure 2b, is used for reinforcing steel bars, which can be expressed as

(3) $$\sigma_{s}=\left\{\begin{array}{cc} E_{s}{ɛ}_{s}, & {ɛ}_{s}\leq f_{t}/E_{s}\\ f_{y}, & {ɛ}_{s}>f_{t}/E_{s} \end{array}\right.,$$

where *E*_s_ is the elastic modulus of steel bars, *f*_y_ is the yield strength of steel bars, *σ*_s_ and *ɛ*_s_ are the tensile stress and tensile strain in steel bars, respectively.

In the numerical simulation, the reinforcing steel bars is modeled by element LINK8, thus the diameter of steel bars *d*_0_ is not considered. The element LINK8 is a 3D uniaxial tension-compression element with three degrees of freedom at each node: translations in the nodal *x*, *y*, and *z* directions, and no bending or twist loads are considered. The element LINK8 can be defined by a generalized force-strain (*F*_s_-*ɛ*_s_) relationship, which can be evaluated using Equation (3) to reflect the mechanical characteristics of steel bars, i.e.,

(4) $$F_{s}=\left\{\begin{array}{cc} \pi d_{0}^{2}E_{s}{ɛ}_{s}/4, & {ɛ}_{s}\leq f_{t}/E_{s}\\ \pi d_{0}^{2}f_{y}/4, & {ɛ}_{s}>f_{t}/E_{s} \end{array}\right..$$

#### 2.1.3. Steel Bar-Concrete Interface

**Figure 3:**
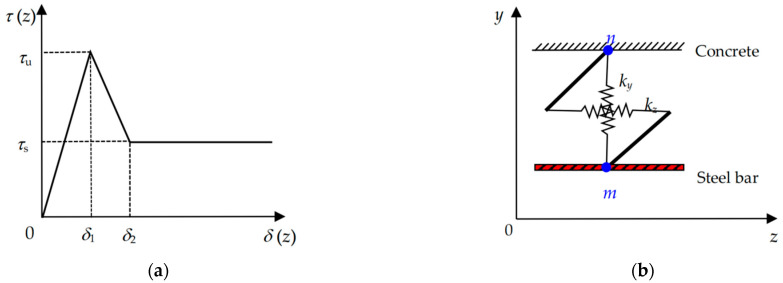
Bond-slip model and double spring elements at steel bar-concrete interface. (**a**) Bond-slip model; (**b**) Double spring elements at steel bar-concrete interface.

As illustrated in Figure 3a, a typical trilinear bond-slip law, which can reasonably describe the interfacial debonding behavior of steel bars embedded in concrete [33,34], is used to represent the bond relationship between the interfacial shear stress *τ* and the interfacial slip *δ* at the steel bar-concrete interface, which can be expressed as

(5) $$\tau =\left\{\begin{array}{cc} \tau_{u}\delta /\delta_{1}, & 0\leq \delta \leq \delta_{1}\\ \left[\left(\tau_{u}\delta_{2}-\tau_{s}\delta_{1}\right)-\left(\tau_{u}-\tau_{s}\right)\delta \right]/\left(\delta_{2}-\delta_{1}\right), & \delta_{1}<\delta \leq \delta_{2}\\ \tau_{s}, & \delta >\delta_{2} \end{array}\right.$$

where *τ*_u_ is the interfacial shear strength, *τ*_s_ is the interfacial frictional strength, *δ*_1_ is the slip corresponding to *τ*_u_, and *δ*_2_ is the slip corresponding to *τ*_s_.

In the numerical simulation, the bond between steel bars and concrete is treated with double spring elements. Each element has a pair of virtual springs with specific mechanical properties, no geometric dimensions, and no masses. As shown in Figure 3b, the elements are defined by two nodes *m* and *n,* which have the same coordinates located in the steel bars and concrete, respectively. Wherein the vertical spring element *k*_y_ in *y*-direction is assumed to be rigid material, which means that no relative vertical displacement along direction *y* occurs between nodes *m* and *n*, while the stiffness of the horizontal spring element *k*_z_ in *z*-direction is defined by the bond-slip constitutive law shown in Equation (5). As in Section 2.1.1, the element COMBIN39 is employed for virtual springs *k*_y_ and *k*_z_. Moreover, it is noteworthy that applying a virtual spring in *x*-direction between nodes *m* and *n* is unessential. According to the loading and geometric symmetry of the specimen, as shown in Figure 1, no relative displacement occurs along the *x*-direction at the bond between steel bars and concrete; therefore, it can be replaced by establishing the *x*-direction boundary constraint conditions for nodes *m* and *n*.

### 2.2. Numerical Modelling

**Figure 4:**
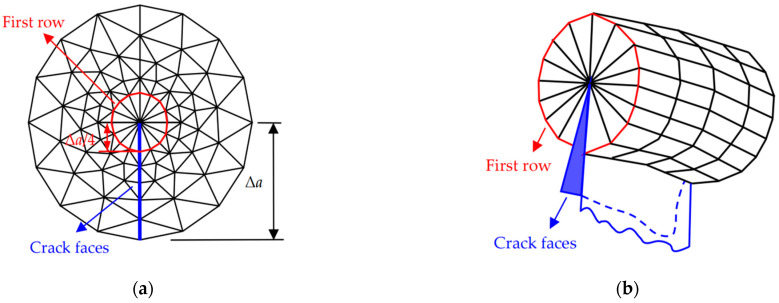
Element meshing at the crack tip: (**a**) cross section; (**b**) 3D.

As shown in Figure 1, the calculation model was formed by a symmetric half width of the RC beam, i.e., *x*∈[0, *B*/2], *y*∈[0, *D*], and *z*∈[−*S*/2, *S*/2]. The material elements for concrete, steel bars, and steel bar-concrete interface were set as Solid95/Combin39, Link8, and Combin39 from the ANSYS material library, respectively. The finite element (FE) meshing was achieved by the LESIZE command, and the resulting mesh from the outer layer to the crack tip zone should be more and more dense. Because high stress gradients exist in the region around the crack tip, fine elements were set up near the crack tip to solve SIFs along the crack tip accurately. As illustrated in Figure 4, the crack tip point is the center of a circle, and the crack propagation length Δ*a* is the radius of the circle. The first row of elements around the crack tip had a radius of Δ*a*/4, and a crack propagation length Δ*a* of (0.5–0.7%) *D* was used.

The boundary constraints for the symmetric simplified RC beam subjecting to three-point bending are the *y*-direction constraints for nodes (*x*, 0, −*S*/2) and (*x*, 0, *S*/2), *x*-direction constraints for nodes (0, *y*, *z*), and *z*-direction constraints for nodes (*x*, *D*, 0), where *x*∈[0, *B*/2], *y*∈[0, *D*], *z*∈[−*S*/*2*, *S*/2], *a*(*x_i_*) is the crack length within the longitudinal section (*x_i_*, *y*, *z*) perpendicular to *x*-coordinate at *x* = *x_i_*. The beam is subjected to three-point bending, and the external load is applied in the vertical direction with the displacement-controlled loading scheme.

**Figure 5:**
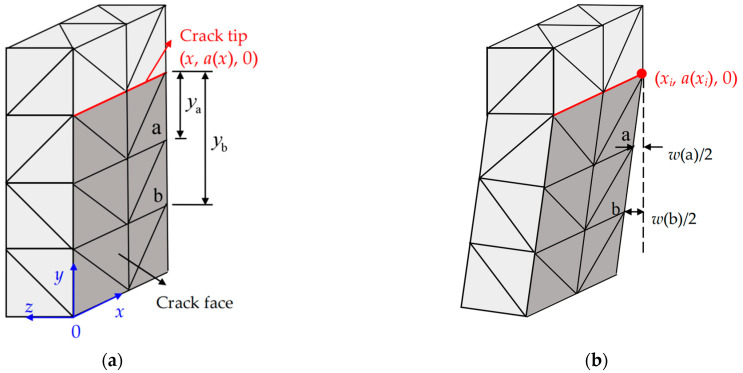
Displacement extrapolation (DE) method: (**a**) Before loading; (**b**) After loading.

The model of an RC beam with a given crack length *a*(*x*) can be simulated by static nonlinear analysis under a specific displacement loading *δ*. Afterwards, compute and output the crack mouth opening displacement *CMOD*, the external load *P*, the bond stress of the steel bar-concrete interface *τ*(*z*), and the SIF at the crack tip (*x*, *a*(*x*), 0), which can be represented by *K*_e_(*x*, *a*) in the result file. Herein, *K*_e_(*x_i_*, *a*) is calculated through the displacement extrapolation (DE) method shown in Figure 5, i.e., ① Select two nodes on the crack faces near the crack tip (*x_i_*, *a*(*x_i_*), 0) along (*x_i_*, *y*, 0), and compute the crack opening displacements along *z*-coordinate *w*(a) and *w*(b) at nodes a and b, respectively; ② Solve the SIFs $K_{I}^{a}$ and $K_{I}^{b}$ at nodes a and b with the relationship between the crack opening displacements *w* and the SIFs shown in Equations (6) and (7), respectively, where *G* is the shear modulus of concrete, *κ* is the Kappa parameter, and *y*_a_ and *y*_b_ are the distances from nodes a and b to the crack tip (*x_i_*, *a*(*x_i_*), 0), respectively; ③ Calculate *K*_e_(*x_i_*, *a*) by linear interpolation with Equation (8).

(6) $$K_{I}^{a}=\frac{2G}{\left(1+\kappa \right)}\sqrt{\frac{2}{y_{a}}}\;w\left(a\right)$$

(7) $$K_{I}^{b}=\frac{2G}{\left(1+\kappa \right)}\sqrt{\frac{2}{y_{b}}}\;w\left(b\right)$$

(8) $$K_{e}\left(x_{i},a\right)=\frac{y_{a}K_{I}^{b}-y_{b}K_{I}^{a}}{y_{a}-y_{b}}$$

### 2.3. Numerical Process

Based on the initial fracture toughness criterion proposed by Wu et al. [35], for quasi-brittle materials, the crack propagates when the SIF at the crack tip *K*_e_ reaches the initial fracture toughness $K_{\mathrm{IC}}^{\mathrm{ini}}$ of the material. Considering the 3D fracture process of a notched lightly reinforced beam, the crack propagation criterion can be further rewritten as

(9) $$K_{e}^{\max}\left(x,a\right)=K_{\mathrm{IC}}^{\mathrm{ini}},$$

where $K_{e}^{\max}$(*x*, *a*) is the maximum value of *K*_e_ (*x*, *a*), and *K*_e_ (*x*, *a*) is the SIF at the crack tip (*x*, *a*(*x*), 0), which is equal to the sum of the SIFs caused by the applied load *P*, the cohesive stress *σ*_c_ acting on the FPZ of concrete, and the constraint shear stress *τ* produced by the steel bars to concrete during the fracture process. Therefore, the crack propagation criterion shown in Equation (9) can be formulated as follows:

(10) $${\left[K_{I}^{P}\left(x,a\right)+K_{I}^{\sigma }\left(x,\Delta a\right)+K_{I}^{\tau }\left(x,a\right)\right]}^{\max}=K_{\mathrm{IC}}^{\mathrm{ini}}.$$

**Figure 6:**
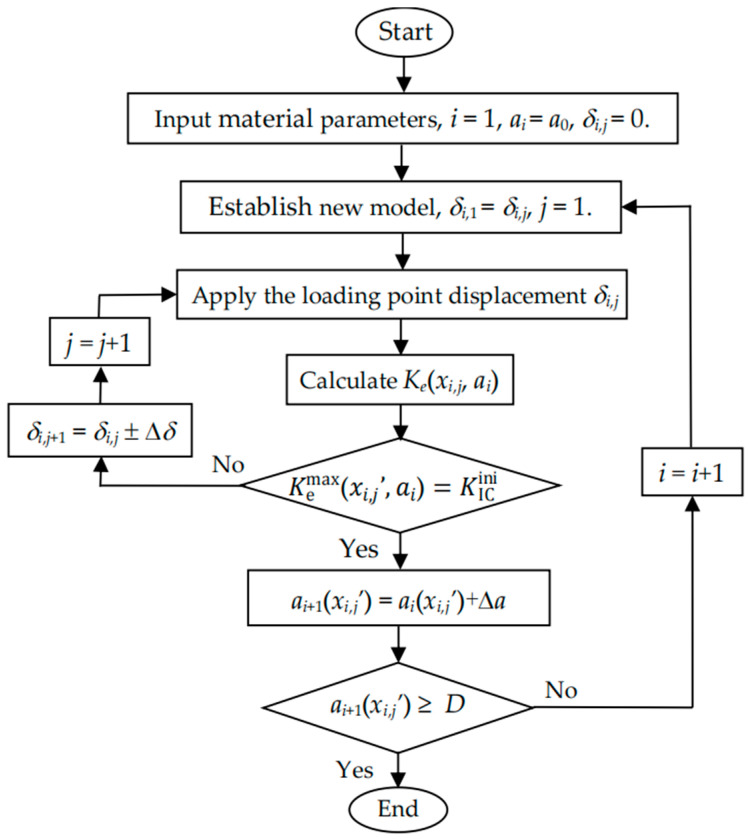
Calculation procedure for crack propagation process of lightly RC beams.

Based on the above crack propagation criterion and the proposed numerical method, the calculation process as shown in Figure 6 can be automatically performed using the ANSYS program written by the ANSYS parametric design language (APDL), so as to complete the numerical simulation of the whole 3D crack propagation process in RC beams. The iteration analysis in the calculation procedure are controlled by the crack length *a*(*x*) and the loading point displacement *δ*. The detailed calculation steps are summarized as follows:

1. Input the specimen dimensions of the RC beam (*S*, *D*, *B*, *d*_0_, *a*_s_, and *a*_0_) and the material parameters (elastic modulus of concrete, *E*_c_, *f*_t_, *G*_F_, $K_{\mathrm{IC}}^{\mathrm{ini}}$, *E*_s_, *f*_y_, *τ*_u_, *τ*_s_, *δ*_1_ and *δ*_2_). Set the iteration variables *i* and *j*, which were controlled by the crack length and the loading point displacement, respectively. Set the initial values, i.e., *i* = 1, *j* = 1, *a_i_* = *a*_0_ and *δ_i,j_* = 0.
2. Establish an FEM model for a three-point bending RC beam with crack length *a_i_*. Set the initial values, i.e., *δ_i,_*_1_ = *δ_i,j_* and *j* = 1.
3. Apply the loading point displacement *δ_i,j_*. Calculate the SIFs at all nodes along the crack tip *K*_e_ (*x_i,j_*, *a_i_*) and obtain its maximum value $K_{e}^{\max}\left(x_{i,j}’,a_{i}\right)$.
4. Verify if the equation $K_{e}^{\max}\left(x_{i,j}’,a_{i}\right)=K_{\mathrm{IC}}^{\mathrm{ini}}$ is satisfied. If $K_{e}^{\max}\left(x_{i,j}’,a_{i}\right)=K_{\mathrm{IC}}^{\mathrm{ini}}$, the crack propagates at node *x_i,j_’*, and the new crack length at node *x_i,j_’* becomes *a_i_*_+1_(*x_i,j_’*) = *a_i_*(*x_i,j_’*)+Δ*a*, where Δ*a* is the specified increment of the crack length. Otherwise, adjust the loading point displacement as *δ_i,j_*_+1_ =*δ_i,j_* ± Δ*δ*, where Δ*δ* is the specified increment of the loading point displacement. Set *j* as *j* = *j* + 1 and go back to step 3.
5. Check if the crack tip reaches the specimen boundary. If *a_i_*_+1_(*x_i,j_’*)≥*D*, the numerical process terminates. Otherwise, the crack continues to propagate; set *i* as *i* = *i* + 1, and go to step 2.

## 3. Experimental Verification

**Figure 7:**
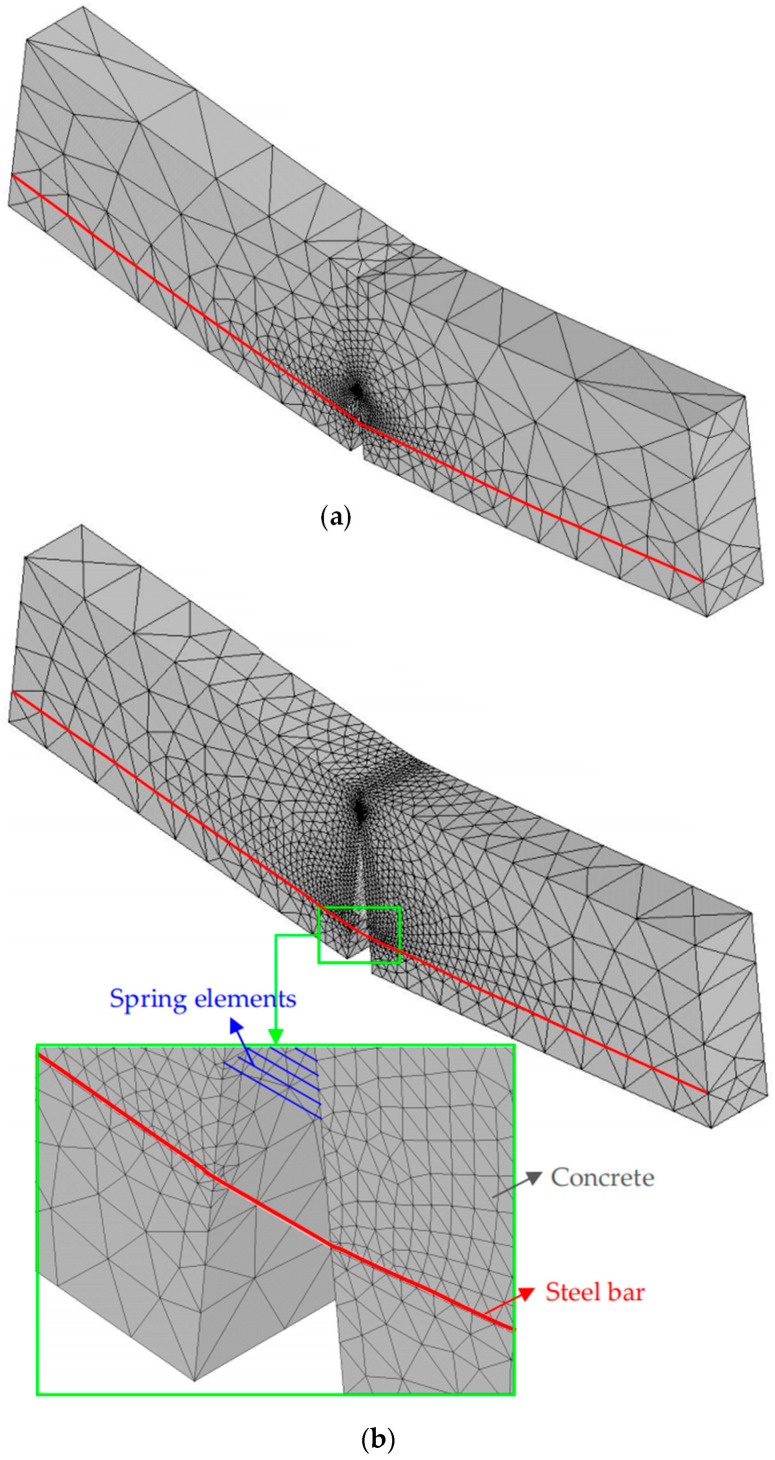
Deformation of different fracture stages for specimen RC203: (**a**) the initial crack starts to propagate; (**b**) the steel bars yields.

**Figure 8:**
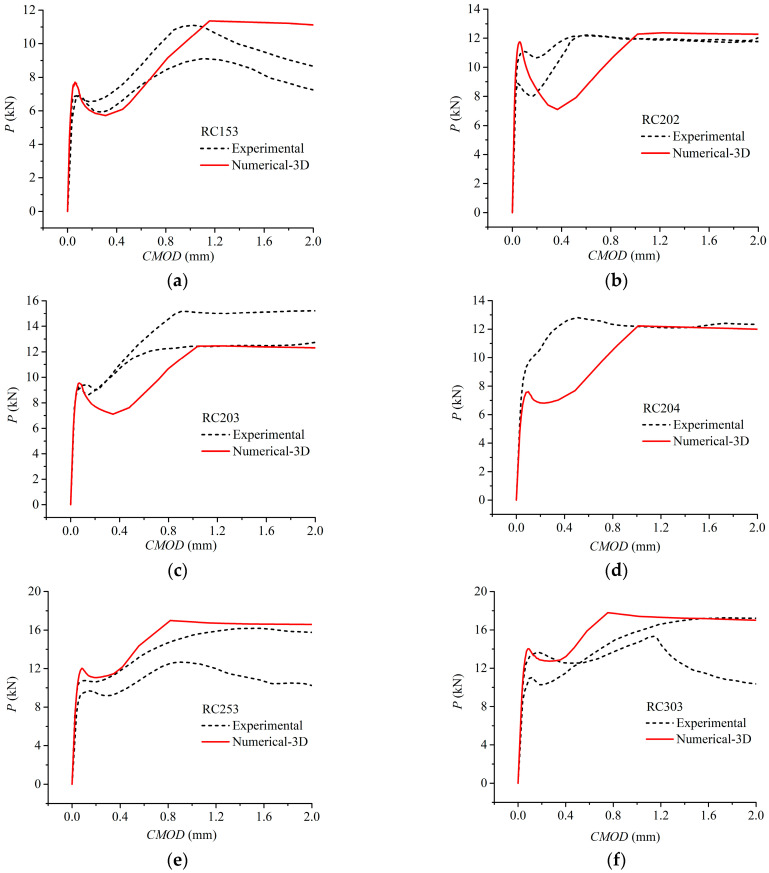
Comparison of numerical and experimental *P*-*CMOD* curves for lightly RC beams: (**a**) RC153; (**b**) RC202; (**c**) RC203; (**d**) RC204; (**e**) RC253; (**f**) RC303.

To verify the validity of the proposed 3D FE model, a comparison of *P*-*CMOD* curves acquired from experimental results on notched TPB lightly RC beams conducted by Yi [10] is demonstrated. In the tests, *E*_c_ = 25.92 GPa, *f*_t_ = 2.25 MPa, *G*_F_ = 135 N/m, and *E*_s_ = 210 GPa. Detailed information regarding dimension and material parameters are listed in Table 1 and Table 2, where $P_{\mathrm{RC}}^{\mathrm{ini}}$ is the initial cracking load of RC beam, $P_{C}^{\mathrm{ini}}$ is the initial cracking load of plain concrete beam, and $K_{\mathrm{IC}}^{\mathrm{ini}}$ is the initial fracture toughness of concrete. In the fracture test, all beams failed after the steel bars yielded and the fictitious cracks propagated to the specimen boundaries without other new cracks involved, and the interfacial debonding behavior occurred during the fracture process. According to the numerical simulation shown in Figure 6, input the dimension and material parameters (*S*, *D*, *B*, *d*_0_, *a_s_*, *a*_0_, $K_{\mathrm{IC}}^{\mathrm{ini}}$, *f*_y_, *τ*_u_, *τ*_s_, *δ*_1_ and *δ*_2_) listed in Table 1 and Table 2, and the numerical results obtained from the proposed calculation procedure were validated against the experimental results, as depicted in Figure 7 and Figure 8.

Figure 7 shows the deformation evolution organized by the 3D FE model for specimen RC203 at different fracture stages, where Figure 7a presents the stage when the initial crack starts to propagate and Figure 7b presents the stage when the steel bars yield. It can be seen that when the steel bars yield, the crack tip is already close to the specimen boundary. Figure 8 illustrates the comparison between the numerical *P*-*CMOD* curves calculated by the proposed 3D model and the test curves, and it shows a reasonable agreement between them for different beam heights *D* and initial crack length ratios *a*_0_/*D*. Figure 8 also indicates that every predicted *P*-*CMOD* curve has two peak loads, *P*_1,max_ and *P*_2,max_, and its shape is characterized by three ascending stages and two descending stages. The comparison between the numerical and experimental results of the initial cracking load in RC beams $P_{\mathrm{RC}}^{\mathrm{ini}}$ and two peak loads are shown in Table 3. It is seen that the average relative absolute errors for $P_{\mathrm{RC}}^{\mathrm{ini}}$, *P*_1,max_ and *P*_2,max_ are 7.18%, 12.60%, and 9.01%, respectively, which indicates that the predicted loads are in reasonably good agreement with the experimental ones. Therefore, the presented 3D numerical method can evaluate the load-carrying capacity of lightly RC beams with reasonable accuracy.

## 4. Analysis and Discussion

### 4.1. Fracture Characteristics of Lightly RC Beam

**Figure 9:**
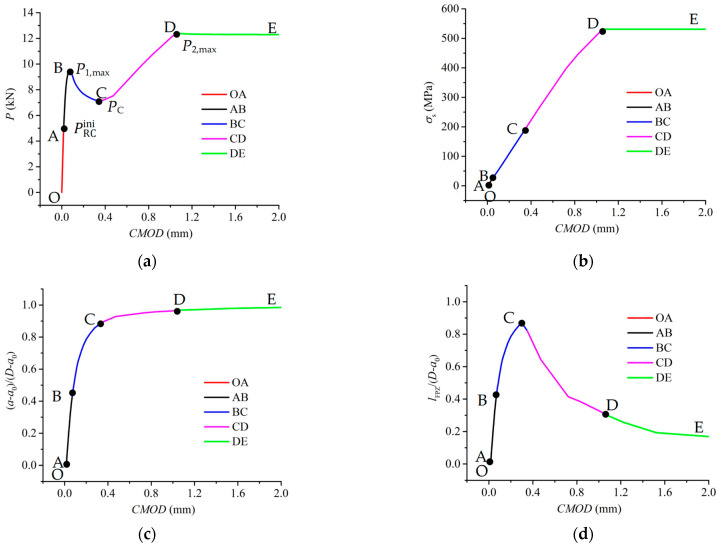
Crack propagation process for specimen RC203: (**a**) *P*-*CMOD* curve, (**b**) *σ*_s_-*CMOD* curve, (**c**) (*a* − *a*_0_)/(*D* − *a*_0_) vs. *CMOD* curve, and (**d**) *l*_FPZ_/(*D* − *a*_0_) vs. *CMOD* curve.

**Figure 10:**
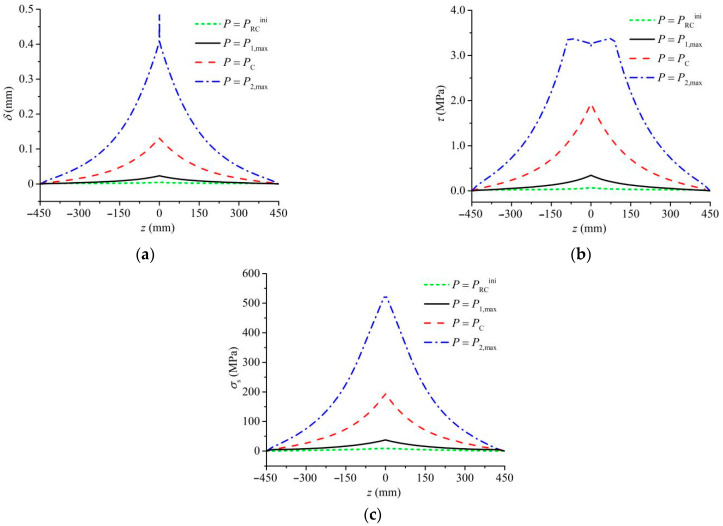
Distribution of (**a**) interfacial slip, (**b**) interfacial shear stress, and (**c**) reinforcement stress for beam RC203.

To evaluate the fracture characteristics of lightly RC beams under three-point bending during the crack propagation process, the test specimen RC203 from Yi [10] is selected as an example. Utilizing the proposed method, various parameters such as external load *P*, steel bars stress *σ*_s_, crack length *a*, FPZ length *l*_FPZ_, interfacial slip *δ*, and interfacial shear stress *τ* were obtained throughout the failure process, as depicted in Figure 9 and Figure 10.

Figure 9 illustrates the variation curves of the external load *P*, the reinforcement stress at the cracked faces *σ*_s_(z = 0), the normalized crack growth length (*a* − *a*_0_)/(*D* − *a*_0_), and the normalized FPZ length *l*_FPZ_/(*D* − *a*_0_) versus the cracking mouth opening displacement *CMOD* for specimen RC203. It can be seen that the fracture process consists of the following five stages:

- Stage I (segment OA): linear increase in *P* before reaching the initial load $P_{\mathrm{RC}}^{\mathrm{ini}}$, with a small value of *σ*_s_(*z* = 0).
- Stage II (segment AB): nonlinear increase in *P* to the first peak load *P*_1,max_, slow increase in *σ*_s_(*z* = 0), and nearly linear growth of (*a* − *a*_0_)/(*D* − *a*_0_), and *l*_FPZ_/(*D* − *a*_0_). That is to say, *P*_1,max_ is determined by the cohesive stress in concrete.
- Stage III (segment BC): decline in *P* from *P*_1,max_ to the minimum load *P*_C_ due to the softening behavior of concrete, significant increases in *σ*_s_(*z* = 0), nonlinear growth of (*a* − *a*_0_)/(*D* − *a*_0_) and *l*_FPZ_/(*D* − *a*_0_).
- Stage IV (segment CD): increase in *P* to the second peak load *P*_2,max_ due to the bridging force of steel bars, increase in *σ*_s_ (*z* = 0) to the yield strength of steel bars *f*_y_, slow growth of (*a* − *a*_0_)/(*D* − *a*_0_), and descend of *l*_FPZ_/(*D* − *a*_0_). Therefore, *P*_2,max_ is controlled by the bridging force of the steel bars.
- Stage V (segment DE): stabilization of *P* as the crack tip approaches the specimen boundary, constant *σ*_s_(*z* = 0), and continued decrease of *l*_FPZ_/(*D* − *a*_0_) until it eventually closes to zero.

Figure 10 illustrates the distributions of the steel bar-concrete interfacial slip *δ*(*z*), the interfacial shear stress *τ*(*z*), and the reinforcement stress *σ*_s_(*z*) of specimen RC203 under four different external loads $P_{\mathrm{RC}}^{\mathrm{ini}}$, *P*_1,max_, *P*_C_, and *P*_2,max_, and it shows that

- When *P* = $P_{\mathrm{RC}}^{\mathrm{ini}}$, *δ*(*z*), *τ*(*z*), and *σ*_s_(*z*) are minimal, making the constraint effect of the steel bars on crack opening negligible;
- When *P* = *P*_1,max_, the values of δ(*z*), τ(*z*), and σs(*z*) near the crack faces (*z* = 0) slightly increase, with the constraint effect of the steel bars on the cracking process still quite small;
- When *P* = *P*_C_, *δ*(*z*), *τ*(*z*), and *σ*_s_(*z*) significantly increase, showing that *δ*(*z*), *τ*(*z*), and *σ*_s_(*z*) increase with the absolute value of *z* decreasing, signifying a substantial enhancement in the constraint effect of steel bars on crack extension;
- When *P* = *P*_2,max_, *δ*(*z* = 0) significantly increases, *τ*(*z*) appears softening behavior near the crack faces (*z* = 0), and *σ*_s_(*z* = 0) reaches the yield strength of steel bars *f*_y_. Therefore, the steel bars have yielded, and the constraint effect of the steel bars on crack propagation reaches its maximum.

Overall, the results indicate that a certain reinforcement slip had appeared during the fracture process, and the two peak loads *P*_1,max_ and *P*_2,max_ are controlled by the cohesive stress in concrete and the bridging force of steel bars, respectively, as reported in the literature [12].

### 4.2. Influence of Specimen Width on Crack Propagation Process

**Figure 11:**
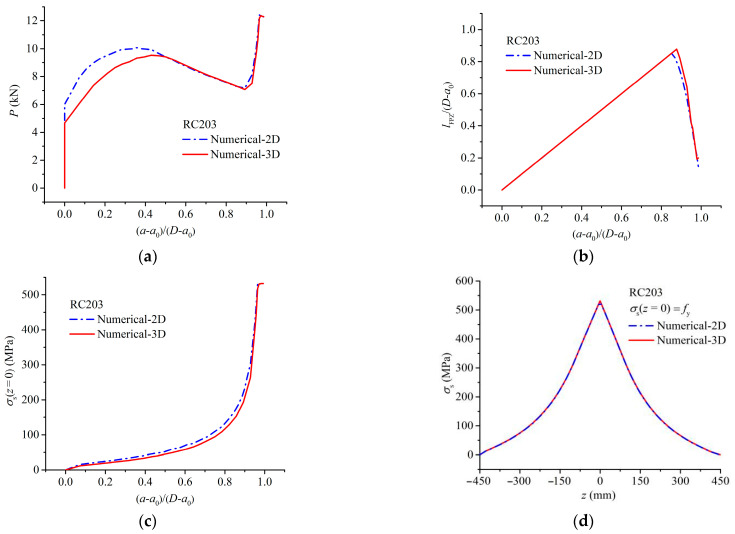
Comparison between 2D and 3D numerical results for specimen RC203: (**a**) *P* vs. (*a*_F_ − *a*_0_)/(*D* − *a*_0_) curve, (**b**) *l*_FPZ_/(*D* − *a*_0_) vs. (*a*_F_ − *a*_0_)/(*D* − *a*_0_) curve, (**c**) *σ*_s_(*z* = 0) vs. (*a*_F_ − *a*_0_)/(*D* − *a*_0_) curve, and (**d**) distribution of reinforcement stress.

Examining specimen RC203 as a case, the influence of specimen width on crack propagation process was discussed by comparing 2D and 3D numerical results, as shown in Figure 11, which includes the curves of the load *P*, the normalized FPZ length *l*_FPZ_/(*D* − *a*_0_), and the steel bars stress at the crack faces *σ*_s_(*z* = 0) versus the normalized crack growth length (*a* − *a*_0_)/(*D* − *a*_0_), and the distribution of reinforcement stress *σ*_s_ along the steel longitudinal direction (*z*-direction) when the steel bars yields. It can be seen that, compared to 3D numerical results, the 2D numerical results exhibit the following distinctions:

1. In the initial loading stage, the load *P*_2D_ obtained by the 2D model significantly exceeds that obtained by the 3D model. As the crack length increases, the discrepancy gradually diminishes. When (*a* − *a*_0_)/(*D* − *a*_0_) surpasses 0.5, the loads *P* obtained by the 2D and 3D models almost converge, as depicted in Figure 11a. This is because in the initial loading stage, the small distance from the steel bars to the crack tip intensifies the local constraint effect of the steel bars on crack opening, amplifying the unevenness of the crack tip SIFs *K*_e_(*x*, *a*) along the width direction (*x*-direction) in 3D modeling. While the 2D model assumes a uniform distribution of *K*_e_ along the width direction. Therefore, when the maximum value of the SIFs at the crack tip $K_{e}^{\max}\left(x,a\right)$ reaches the initial fracture toughness $K_{\mathrm{IC}}^{\mathrm{ini}}$ (i.e., $K_{e}^{\max}\left(x,a\right)=K_{\mathrm{IC}}^{\mathrm{ini}}$) in the 3D model, the average value of *K*_e_ in the 2D model remains smaller than $K_{\mathrm{IC}}^{\mathrm{ini}}$, and a larger load is required in the 2D model. As the crack grows, the influence of the steel bars on the SIF distribution weakens, and the error in the 2D numerical calculation decreases accordingly. Therefore, in the later loading stage, the loads *P* obtained from 2D, and 3D models almost coincide.
2. The maximum normalized FPZ length (*a*_F_ − *a*_0_)/(*D* − *a*_0_) calculated by the 2D model is smaller than that by the 3D model, as shown in Figure 11b. The reason is that the local constraint of steel bars on the crack faces causes *l*_FPZ_ to be unevenly distributed along the beam width. While *l*_FPZ_ calculated by the 2D model represents an average value, resulting in a smaller (*a*_F_ − *a*_0_)/(*D* − *a*_0_);
3. During the crack propagation process, the steel bars stress at the crack faces *σ*_s_(*z* = 0) obtained by the 2D model surpasses the 3D numerical result, as shown in Figure 11c. The reason for this is that in the 2D model, the steel bars is assumed to be a line in the width direction, and the crack opening displacement is assumed to be the same along the beam width. In reality, the constraint of the steel bars on the crack faces concentrates at one point, resulting in the smallest crack opening displacement at the steel bars *δ*_s_ along the beam width. Consequently, the value of *δ*_s_ calculated by the 2D model is larger, leading to a correspondingly larger *σ*_s_(*z* = 0);
4. The distributions of steel bars stress along the steel bars longitudinal direction *σ*_s_(*z*) calculated by the 2D and 3D models are the same when the steel bars yields, as shown in Figure 11d, owing to that *σ*_s_(*z*) mainly depends on the bond-slip relationship between the steel bars and concrete.

**Figure 12:**
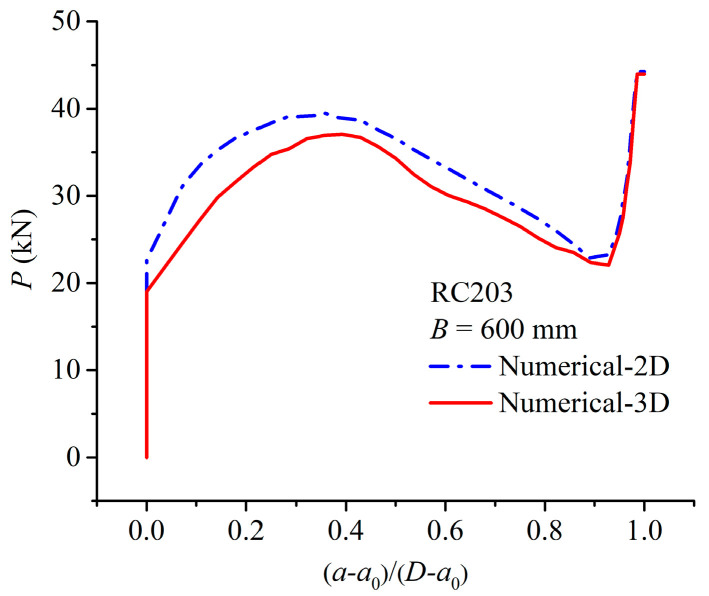
Comparison between 2D and 3D numerical results for specimen RC203 with B = 600 mm.

To further investigate the effect of specimen width on numerical results, both 2D and 3D models were employed to compute load *P* vs. the normalized crack growth length (*a* − *a*_0_)/(*D* − *a*_0_) curve for an exemplary beam with a width of 600 mm based on specimen RC203, as shown in Figure 12. A comparison of Figure 11a and Figure 12 shows that the first intersection point of the result curves from the 2D and 3D models changes from 0.5 to 0.9, while the calculated ultimate loads remain consistent. In other words, as the specimen width increases, it extends the influence on crack propagation along a greater portion of the ligament length. However, this increase in specimen width does not impact the bearing capacity of lightly RC beams.

In summary, by comparing 2D and 3D results, it can be seen that although the overall influence of specimen width on crack propagation process is relatively minor, noticeable differences, particularly in the early stage of cracking, are evident. That is to say, for lightly RC beams subjecting to static loading, compared to the 2D model, using the 3D model to simulate the crack propagation process is more accurate, but the calculated ultimate loads are almost the same. However, for beams with adequate reinforcement or stirrups or beams subjecting to dynamic loading, the situation may be different, which requires further research. In practical engineering applications, an appropriate calculation model should be selected based on the actual circumstances.

## 5. Conclusions

To study the influence of specimen width on the crack propagation process, a 3D finite element method was utilized to simulate the entire fracture process of lightly RC beam. It is assumed that the crack propagates when the maximum SIF at the crack tip along the beam width reaches the initial fracture toughness of concrete. In this method, different nonlinear spring elements were defined to characterize the softening behavior of concrete and the debonding process of the steel-concrete interface, based on the concrete softening constitutive law and the steel bar-concrete interfacial bond-slip relation, respectively. The fracture characteristics such as *P*-*CMOD* curve, reinforcement stress *σ*_s_(*z*), and steel bar-concrete interfacial shear stress *τ*(*z*) could be obtained. The validity of this numerical method was verified by comparing the *P*-*CMOD* curves calculated by the presented method and from experimental data obtained from the literature. Finally, the influence of specimen width on the crack propagation process was analyzed. The following conclusions can be drawn:

1. In the initial loading stage, the external load *P* obtained from the 2D model is significantly larger than the result from the presented 3D model; therefore, the influence of specimen width on the fracture process cannot be ignored. In the final loading stage, the two models yield almost the same results.
2. The first peak of the *P*-*CMOD* curve can be attributed to the action of cohesive forces in concrete, while the second peak corresponds to the yielding of steel bars. Before *P* reaches the first peak load *P*_1,max_, the values of steel bars tensile stress *σ*_s_(*z*) and steel bar-concrete interfacial shear stress *τ*(*z*) are relatively small. After *P* reaches the load *P*_C_, the values of *σ*_s_(*z*) and *τ*(*z*) significantly increase. When *P* reaches the second peak load *P*_2,max_, the steel bars yields, and the values of *σ*_s_(*z*) and *τ*(*z*) no longer change.

## Figures and Tables

**Table 1:** Geometric and material parameters for RC beams.

Specimen No.	*S* × *D* × *B* (mm^3^)	*a*_0_ (mm)	*d*_0_ (mm)	*a*_s_ (mm)	$P_{RC}^{ini}$ (kN)	$P_{C}^{ini}$ (kN)	$K_{IC}^{ini}$ (MPa∙m^1/2^)
RC153	600 × 150 × 150	45	6	35	3.64	3.11	0.33
RC202	800 × 200 × 150	40	6	35	5.90	5.38	0.38
RC203	800 × 200 × 150	60	6	35	4.48	4.32	0.39
RC204	800 × 200 × 150	80	6	35	3.50	3.30	0.39
RC253	1000 × 250 × 150	75	8	35	5.95	5.17	0.42
RC303	1200 × 300 × 150	90	8	35	7.73	6.92	0.51

**Table 2:** Constitutive parameters of steel bar-concrete interface.

Steel Bar Diameter *d*_0_ (mm)	*f*_y_ (MPa)	*δ*_1_ (mm)	*τ*_u_ (MPa)	*δ*_2_ (mm)	*τ*_s_ (MPa)
6	530	0.23	3.39	0.64	3.09
8	385	0.20	3.47	0.70	2.95

**Table 3:** Comparison between numerical and experimental results.

Specimen No.	$P_{RC}^{ini}$ (kN)			*P*_1,max_ (kN)			*P*_2,max_ (kN)		
	Exp.	Num.	Relative Error (%)	Exp.	Num.	Relative Error (%)	Exp.	Num.	Relative Error (%)
RC153	3.64	3.23	−11.3	6.90	7.70	11.6	10.13	11.36	12.1
RC202	5.90	5.72	−3.1	9.87	11.37	15.2	12.28	12.38	0.8
RC203	4.48	4.77	6.5	9.27	9.54	2.9	13.92	12.45	−10.6
RC204	3.50	3.11	−11.1	9.72	7.61	−21.7	12.78	12.22	−4.4
RC253	5.95	5.38	−9.6	10.79	12.02	11.4	14.52	16.98	16.9
RC303	7.73	7.85	1.6	12.44	14.03	12.8	16.30	17.80	9.2
Average			7.18			12.60			9.01

## Data Availability

The original contributions presented in the study are included in the article, further inquiries can be directed to the corresponding author.
